# Rat Chondrocyte Inflammation and Osteoarthritis Are Ameliorated by Madecassoside

**DOI:** 10.1155/2020/7540197

**Published:** 2020-02-01

**Authors:** Safwat Adel Abdo Moqbel, Yuzhe He, Langhai Xu, Chiyuan Ma, Jisheng Ran, Kai Xu, Lidong Wu

**Affiliations:** Department of Orthopedic Surgery, The Second Affiliated Hospital, School of Medicine, Zhejiang University, Hangzhou 310000, China

## Abstract

As a joint disease, osteoarthritis (OA) is caused by the breakdown of subchondral bone and cartilage damage. Inflammatory factors, such as interleukin- (IL-) 1*β*, mediate the progression of OA. Madecassoside (MA), a triterpenoid component derived from the gotu kola herb (*Centella asiatica*), exhibits various pharmacological effects, including antioxidative and anti-inflammatory properties. In the present study, the protective effects and possible mechanism of MA on the treatment of OA were investigated. MA was demonstrated to significantly suppress the IL-1*β*-induced overexpression of matrix metalloproteinase- (MMP-) 3, MMP-13, inducible nitric oxide synthase (iNOS), and cyclooxygenase-2 (COX-2) and to decrease the IL-1*β*-induced degradation of type II collagen and sox9. Additionally, MA was able to reduce the IL-1*β*-induced phosphorylation of p65 in osteoarthritic chondrocytes. Furthermore, in a rat OA model, MA prevented cartilage degeneration and reduced the OARSI score in the MA-treated group compared with the OA group. The present study showed that MA suppresses the nuclear factor-*κ*B signaling pathway, reducing IL-1*β*-induced chondrocyte inflammation, which indicates the therapeutic potential of MA in patients with OA.

## 1. Background

Osteoarthritis (OA) is a musculoskeletal disorder in the aged population [1]. OA is the most common cause of disability among aging patients in the US [2]; it is characterized by a gradual loss of articular cartilage, joint stiffness, and chronic pain and severely affects quality of life. A number of factors, such as sex, weight, and trauma, may increase the risk of developing OA [3], which may ultimately result in joint degeneration. Chondrocytes are responsible for maintaining the normal synthesis and renewal of the cartilage matrix, and this function may be retarded in the inflammatory microenvironment [4]. Previous studies showed the impact of inflammatory mediators on the pathological mechanism of OA. Among these inflammatory mediators, interleukin- (IL-) 1*β* has been recognized as an important instigator of the pathophysiological development of OA [5]. It is able to stimulate chondrocyte-induced activation of the nuclear factor- (NF-) *κ*B pathway, resulting in the upregulation of cartilage degrading enzymes such as matrix metalloproteinase- (MMP-) 1, MMP-3, and MMP-13 [6, 7]. Furthermore, IL-1*β* alters the expression of chondrocyte-specific proteins, including type II collagen, and sox-9 in OA pathogenesis. Following stimulation by inflammatory cytokines in OA, inducible nitric oxide synthases (iNOS) produces large quantities of nitric oxide, resulting in the overproduction of cyclooxygenase2 (COX2) and an increase in the production of prostaglandin E2 (PGE2). PGE2 may increase MMP-13 production, which leads to collagen degradation [8] and the IL-1*β*-induced suppression of the inflammatory response may suppress the development of OA.

Madecassoside (MA) is a bioactive triterpenoid saponin with a molecular weight of 975.12 kDa that is isolated from the gotu kola herb (*Centella asiatica*), Chemical Abstracts Service no. FY1352B1209 (C48H78O20). Previous studies have reported that MA exhibits antioxidant and anti-inflammatory activities [9] and is able to suppress the activation of the NF-*κ*B signaling pathway [10–12]. However, the inhibitory effects of MA on articular cartilage degradation have not been reported yet. Thus, the presented study is aimed at investigating the protective role of MA on IL-1*β*-induced osteoarthritic chondrocytes, in addition to its underlying mechanism.

## 2. Materials and Methods

### 2.1. Chemicals and Materials

Madecassoside was purchased from Nantong Feiyu Biological Technology, Co., Ltd. DMEM, FBS, streptomycin, penicillin, and 0.25% pancreatic enzyme were all obtained from Gibco, NY, USA. Recombinant rat IL-1*β* was purchased from R&D Systems, Abingdon, UK, and collagenase II, DMSO, and BSA were obtained from Sigma-Aldrich, Merck KGaA, MO, USA. The bicinchoninic acid (BCA) assay kit and RIPA buffer were purchased from the Beyotime Institute of Biotechnology, Shanghai, China.

### 2.2. Isolation, Culture, and Treatment of Chondrocytes

Briefly, cartilage was extracted from the hip joints of fifteen 4-week-old male Sprague-Dawley rats weighting (140 ± 10 g). Food and water were routinely provided and were housed at room temperature (23-25°C) with 12 h light/dark cycle under 50-60% relative humidity. The rats were euthanized with 100% CO_2_ with a chamber volume of 20% per minute. In 2-3 min, breathing loss and eye color fading were observed during the procedure. Per sterile requirements, the articular cartilage was isolated and digested using 0.25% pancreatic enzyme for 30 min; to isolate chondrocytes, the tissues were then digested for a further 4 h using DMEM containing 0.2% collagenase at 37°C. The cell suspension was centrifuged to harvest the chondrocytes, which were subsequently cultured in DMEM (10% FBS and penicillin-streptomycin) at 37°C with 5% CO_2_ until confluent; the medium was changed at 24 h intervals. The cells were harvested by trypsinization treated with different concentrations of MA for 1 h then with or without IL-1*β* (10 ng/ml) for a further 24 h or 30 min.

### 2.3. Cell Counting Kit (CCK-8) Analysis

To determine the cytotoxicity of MA on chondrocytes, CCK-8 assay (Nanjing KeyGen Biotech Co., Ltd.) was used according to the manufacturer's protocol. Passage 3 cells in DMEM/F12 were seeded into 96-well plates (4 × 10^3^/well) for 24 h, and the media was then substituted for DMEM/F12 + 0, 25, 50, 100, and 200 *μ*M MA for 24 and 48 h. The media was replaced with fresh media containing 10% CCK-8 solution, and the cells were incubated for 3 h at 37°C. The optical density (OD) was determined, and the experiment was independently repeated three times.

### 2.4. Ribonucleic Acid Isolation and Reverse Transcription-Quantitative (RT-Q) PCR

Chondrocytes were cultured and pretreated with different concentrations of MA, prior to total RNA isolation on ice using TRIzol® reagent (Invitrogen, CA, USA). Total RNA was quantified according to the absorbance at 260 nm (A260)/A280 ratio, which was measured by DeNovix, and RNA was used to synthesize cDNA using PrimeScript™ RT Master Mix Kit purchased from Takara Biotechnology Co., Ltd; the reaction was conducted at 37°C for 15 min, followed by 5 min at 85°C. qPCR was conducted using the Power SYBR® Green master mix (Applied Biosystems, Thermo Fisher Scientific, Inc.), with the StepOnePlus™ Real Time PCR system according to the manufacturer's protocol. Each 10 *μ*l sample contained 5 *μ*l of SYBR® Green, 0.4 *μ*l of each forward and reverse primer, 1 *μ*l of cDNA (10 ng), and 3.2 *μ*l of ddH_2_O. The thermocycling conditions were as follows: denaturation, 95°C × 30 sec, followed by 40 cycles of 95°C × 15 sec → 60°C × 32 sec → 72°C × 1 min → 72°C × 5 min. The primer sequences were as follows: cox-2 F.P.S., 5′-GAGAGATGTATCCTCCCACAGTCA-3′ and R.P.S., 5′ GACCAGGCACCAGACCAAAG-3′; iNOS F.P.S., 5′-CCTACGAGGCGAAGAAGGACAG-3′ and R.P.S., 5′-CAGTTTGAGAGAGGAGGCTCCG-3′; MMP-3 F.P.S. 5′-CAGGCATTGGCACAAAGGTG-3′ and R.P.S., 5′-GTGGGTCACTTTCCCTGCAT-3′; MMP-13 F.P.S., 5′-GCAAACCCTGCGTATTTCCAT-3′ and R.P.S., 5′-GATAACCATCCGAGCGACCTTT-3′; collagen II F.P.S., 5′-GAGTGGAAGAGCGGAGACTACTG-3′ and R.P.S., 5′-GTCTCCATGTTGCAGAAGACTTTCA-3′; sox-9 F.P.S., 5′-CCAGCAAGAACAAGCCACAC-3′, and R.P.S., 5′-CTTGCCCAGAGTCTTGCTGA-3′; and 18S F.P.S. 5′-CCTGAGAAACGGCTACCACA-3′ and R.P.S., 5′-ACCAGACTTGCCCTCCAATG-3′ and 18S was used as the endogenous control. We performed all gene analyses in triplicate. Using the formula 2 − ΔΔCT to analyze the gene expression data [13].

### 2.5. Protein Extraction and Western Blotting

The chondrocytes were divided into eight groups, and whole protein was extracted using 100 *μ*L radioimmunoprecipitation assay lysis buffer, 1% phenylmethylsulfonyl fluoride, and 0.1% phosphorylated proteinase inhibitor (Invitrogen, Thermo Fisher Scientific, Inc.) for 40 min. The proteins were quantified using a BCA assay kit, adjusted to a uniform concentration, and denatured at 100°C for 10 min. Equal volumes of protein were separated by SDS-PAGE with an 8 or 10% gel and electrotransferred onto membranes. After being blocked with 5% BSA (Sigma-Aldrich; Merck KGaA) for 1 h, the membranes were probed with the corresponding primary antibodies against MMP-3 (rabbit, cat. no. #ab52915; Abcam), MMP-13 (rabbit, cat. no. #ab39012; Abcam), iNOS (rabbit, cat. no. #ab3523; Abcam), COX-2 (rabbit, cat. no. #12282; Cell Signaling Technology, Inc.), collagen II (rabbit, cat. no. #ab34712; Abcam), sox9 (rabbit, cat. no. #ab185966Ab; Abcam), NF-*κ*B p65 (rabbit, cat. no. #4764S; Cell Signaling Technology, Inc.), pp65 ((Ser536) (rabbit, cat. no. #3031; Cell Signaling Technology, Inc.), NF-*κ*B inhibitor *α* (I*κ*B-*α*) (rabbit, cat. no. #4812; Cell Signaling Technology, Inc.), and p-I*κ*B-*α* ((Ser32); 14D4; rabbit, cat. no. #2859; Cell Signaling Technology, Inc.) at 4°C overnight. GAPDH (rabbit, cat. no. #ab70699; Abcam) or *β*-actin (mouse, cat. no. #ab8226; Abcam) were used as the endogenous controls. All antibodies were used at 1 : 1000 dilution. After being washed three times with TBS-T, the membranes were probed with a secondary antibody (cat. no. A0208; Beyotime Institute of Biotechnology) for 2 h at room temperature. After a further three washing, the protein bands were visualized using an ECL kit (Immobilon Western Chemiluminescent HRP Substrate; cat. no. WBKLS0050; Merck KGaA) and quantified using a Bio-Rad ChemiDoc system (Bio-Rad Laboratories, Inc.).

### 2.6. Safranin O Staining of Chondrocytes

The chondrocytes were seeded into 12 plates (5 × 10^5^ cells/well) and treated with MA. The cells were subsequently fixed using 4% paraformaldehyde solution for 15 min and then stained with safranin O for 7 min at room temperature. Cells were then washed three times in PBS, and images were captured using a gross camera and a microscope. The experiment was independently repeated three times.

### 2.7. Immunofluorescence Staining

Chondrocytes were seeded in coverslips for 12 h to investigate the key protein of the NF-*к*B pathway. The cells were pretreated with MA for 1 h and then stimulated with 10 ng/ml IL-1*β* for 30 min. Cold methanol was used to fix the cells for 20 min and then permeabilized for 15 min with 0.3% *v*/*v* Triton X-100. After blocking with 5% BSA for 1 h at room temperature, cells were incubated with primary antibody against p65 at 4°C overnight. After washing, the cells were incubated with secondary antibody (AB) conjugated with fluorescein-isothiocyanate (Alexa Fluor 555-labeled Donkey Anti-Rabbit IgG (H+L) (cat. no. A0453; Beyotime Institute of Biotechnology) in the dark for 1 h and the DAPI staining for 5 min. We used a Leica fluorescence microscope to view the results.

### 2.8. Animal Experiments

A total of 21 male Sprague-Dawley rats (6-week-old; 180–240 g) were divided into three groups to investigate the role of MA on OA *in vivo*. Food and water, light/dark cycle, and housing condition were the same as mentioned above. Experimentally, the rats received a medial meniscus resection to induce OA. Briefly, the rats were anesthetized with a peritoneal injection of pentobarbital (40 mg/kg), the knee joints were opened, and the medial meniscus was carefully resected in the OA- and MA-treated groups, without any articular cartilage or ligament damage. The sham group received an arthrotomy without medial meniscus transection.

After one week, 200 *μ*M of MA was intra-articularly injected to the knee joints of the rats, once a week. After four weeks, the rats were euthanized with 100% CO_2_ as described in the chondrocyte culture section, and the knee joint specimens were fixed in 4% paraformaldehyde solution. After decalcification for two months with 10% EDTA, the specimens were dehydrated using ascending of ethanol series and embedded in paraffin blocks. The study protocol was approved by the Ethics committee of The Second Affiliated Hospital, School of Medicine, Zhejiang University (Hangzhou, China; approval no. 2015-107).

### 2.9. Histological Analysis

Using a microtome, the knee joint specimens were cut into 5 mm slices, deparaffinized with xylene, and subsequently rehydrated using a graded ethanol series. The sections were then stained with safranin O/Fast Green and hematoxylin-eosin staining. Histological evaluation was conducted by three individuals using OARSI scoring system, applied in a blinded manner. According to the loss of safranin O, structural changes, and erosion area, OARSI scoring system can be used to evaluate the osteoarthritic cartilage.

### 2.10. Immunohistochemical Analysis

Immunohistochemical staining was used to assess OA in the sagittal sections of the knee joint from each group. The knee sections were prepared as described in the histological analysis and then subjected to antibody against MMP-3 (rabbit, cat. no. #ab52915; Abcam), MMP-13 (rabbit, cat. no. #ab39012; Abcam), and COX-2 (rabbit, cat. no. #12282; Cell Signaling Technology, Inc.).

### 2.11. Statistical Analysis

The data are presented as the means ± standard deviation. One-way analysis of variance followed by Tukey's post hoc test were used to determine the statistical significance between test groups (Prism 7.00; GraphPad Software, Inc.,), and *P* < 0.05 was considered to indicate a statistically significant difference.

## 3. Results

### 3.1. Effects of MA on Rat Chondrocyte Viability

**Figure 1:**
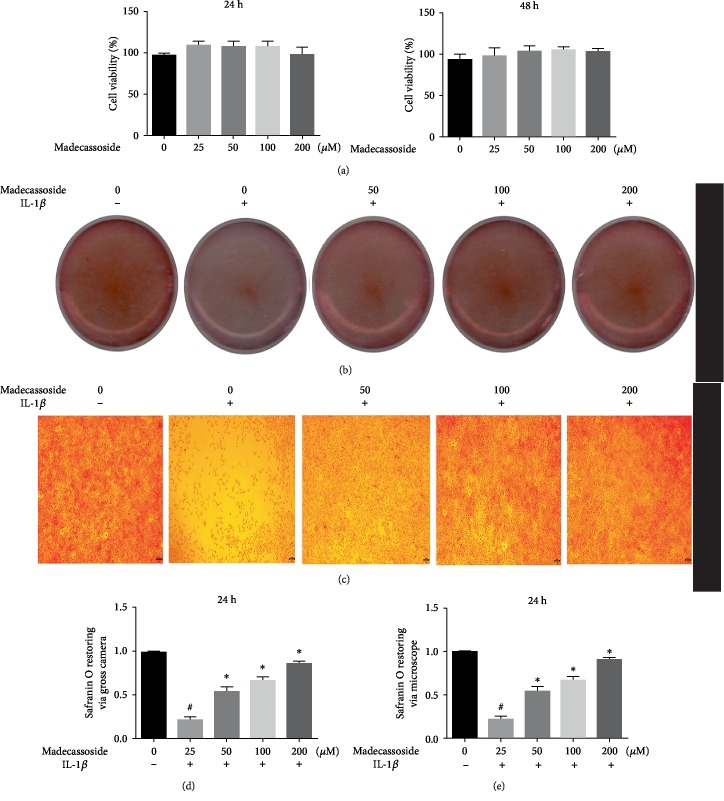
Effect of MA on the viability of chondrocytes. (a) Various concentrations of MA were added to chondrocytes for 24 and 48 hours, and the Cell Counting Kit-8 was used to evaluate the influences of MA on rat chondrocyte viability. *N* = 3; the data are expressed as the mean ± standard deviation. (b) and (d) Gross camera image of safranin O staining of IL-1*β*-induced chondrocytes subjected to different concentrations of MA and its quantitative analysis for 24 h. *N* = 3; data are expressed as the mean ± standard deviation. (c) and (e) Effects of MA on IL-1*β*-stimulated chondrocytes and its quantitative analysis for 24 h by a microscope. MA: madecassoside; IL: interleukin.

The cytotoxic effects of MA on chondrocyte viability were determined using CCK-8 assay. As shown in Figure 1(a), ≤200 *μ*M MA had no obvious inhibitory effects on rat chondrocyte viability at 24 and 48 h. Thus, concentrations of 50, 100, and 200 *μ*M were selected to investigate the anti-inflammatory effects *in vitro*.

### 3.2. MA Reduced the Expression Levels of MMP-3, MMP-13, iNOS, and COX-2 and Upregulated the Cartilage-Specific Proteins

**Figure 2:**
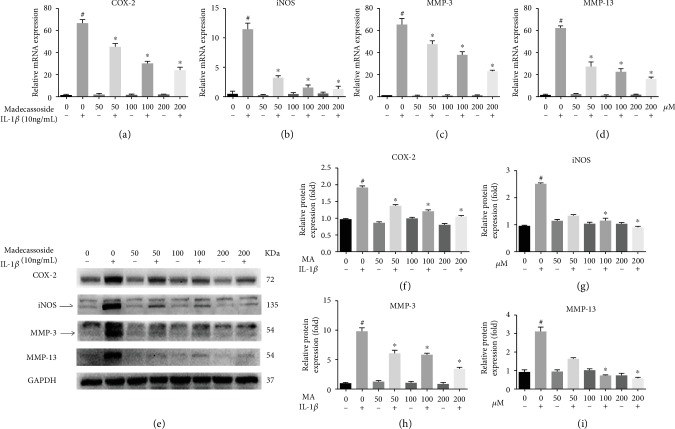
MA suppresses the expression levels of MMP-3 and MMP-13, COX-2, and iNOS in IL-1*β*-induced rat osteoarthritic chondrocytes. MA-pretreated chondrocytes were treated with (10 ng/ml) IL-1*β* for 24 hours. The expression of MMPs and inflammatory genes in rat chondrocytes was evaluated at the mRNA level using reverse transcription-quantitative PCR (a)–(d), and western blotting was used to evaluate protein expression levels (e)–(i). *N* = 3; data are expressed as the mean ± standard deviation. ^#^*P* < 0.05 vs. sham group. ^∗^*P* < 0.05 vs. IL-1*β* group. MMP: matrix metalloproteinase; MA: madecassoside; COX-2: cyclooxygenase-2; iNOS: inducible nitric oxide synthase; IL: interleukin.

**Figure 3:**
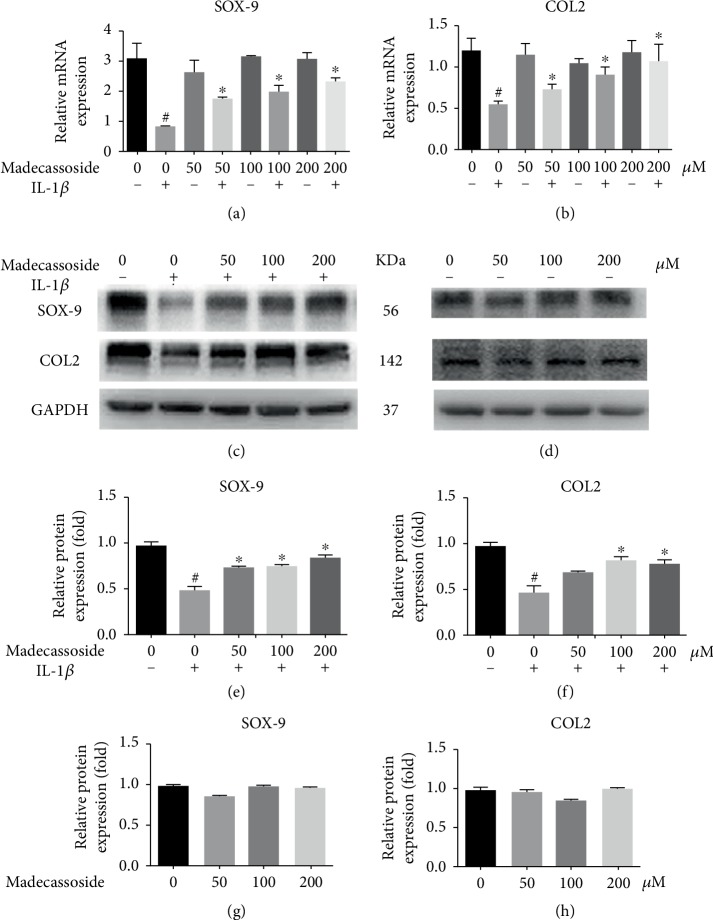
MA upregulates the expression levels of chondrocyte-specific protein. Three groups of MA-pretreated chondrocytes were incubated with and without 10 ng/ml IL-1*β*, and one group stimulated with IL-1*β* in the absence of MA for 24 hours. qRT-PCR at the mRNA level for sox-9 and collagen II (a) and (b). Western blotting and quantitative analysis for SOX-9 and COL II in the presence of IL-1*β* (c), (e), and (f), and the images (d), (g), and (h) in the absence of IL-1*β*. *N* = 3; data are expressed as the mean ± standard deviation. ^#^*P* < 0.05 vs. sham group. ^∗^*P* < 0.05 vs. IL-1*β* group. COL II: collagen II; MA: madecassoside; SOX-9: transcription factor SOX-9; IL: interleukin.

The role of MA on the expression of MMP-3, MMP-13, iNOS, COX-2, sox-9, and collagen II in IL-1*β*-induced chondrocytes was investigated using RT-qPCR and western blotting. The RT-qPCR results revealed that at the mRNA level, all concentrations of MA decreased the IL-1*β*-induced upregulation of inflammatory genes and MMPs and without any significant difference between MA-treated groups in the absence of IL-1*β* and negative control group (Figures 2(a)–2(d)). Additionally, western blot analyses showed that the protein expression levels of COX-2, iNOS, MMP-3, and MMP-13 were also increased in the IL-1*β* group and then were decreased in MA groups, and without any obvious difference between MA without IL-1*β* groups and the negative control group (Figure 2(e)). RT-qPCR and western blotting also showed that 50 *μ*M MA increased the expression levels of sox9 and collagen type II (Figures 3(a)–3(c)), without any obvious differences between MA-treated group in the absence of IL-1*β* and the control group (Figures 3(a), 3(b), and 3(d)). In addition, the glycosaminoglycan is detectable by safranin O staining, which is one of the cartilage matrix components. MA treatment was able to restore the fading of safranin O staining in IL-1*β*-induced rat chondrocytes (Figures 1(b) and 1(c)). Collectively, the data indicated that MA protected rat chondrocytes by promoting chondrocyte-specific protein expression and suppressing the upregulation of matrix-degrading genes.

### 3.3. MA Suppresses IL-1*β*-Induced Stimulation of the NF-*κ*B Signal Pathway in Rat Chondrocytes

**Figure 4:**
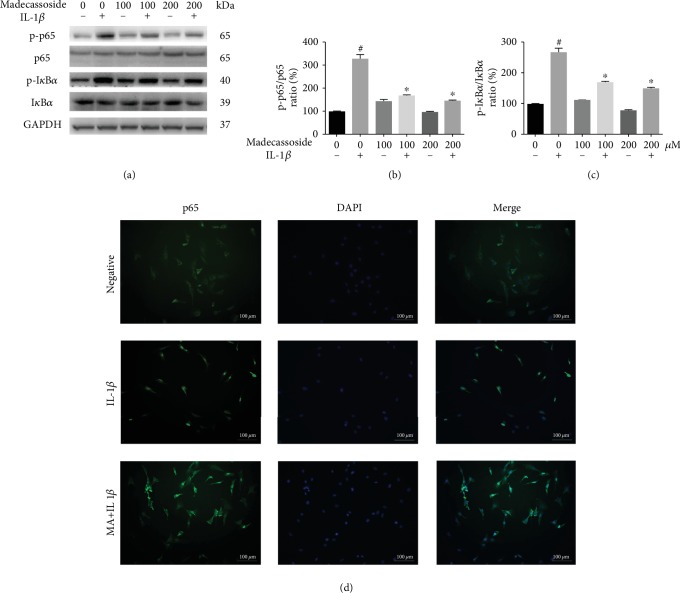
MA inhibits the IL-1*β*-induced activation of pp65/p65 and p-I*κ*B*α*/I*κ*B*α* in rat chondrocytes. Chondrocytes were treated with various concentrations of MA for 1 hour and subsequently stimulated with 10 ng/ml IL-1*β* for 30 min. (a) Western blot analysis and (b) and (c) quantitative analysis relevant to p-p65/p65 and p-I*κ*B*α*/I*κ*B*α*. *N* = 3; data are expressed as the mean ± standard deviation. (d) The immunofluorescence microscopic analysis was used to detect the nuclear translocation of p65. Green, p65; Blue, DAPI. Scale bar = 100 *μ*M. ^#^*P* < 0.05 vs. sham group. ^∗^*P* < 0.05 vs. IL-1*β* group. I*κ*B*α*: NF-*κ*B inhibitor *α*; IL: interleukin.

The possible mechanism of MA was explored by investigating the NF-*κ*B signaling pathway, which protects the cartilage against degradation. The activational statuses of phospho-p65, p65, NF-*κ*B inhibitor *α* (I*κ*B*α*) and p-I*κ*B*α* were investigated by western blotting. This revealed that cells pretreated with 100 and 200 *μ*M MA possessed significantly decreased levels of phospho-p65 and p-I*κ*B*α* induced by IL-1*β* activation; furthermore, MA has no obvious alteration to NF-*κ*B signaling pathway in absence of IL-1*β* (Figure 4(a)). MA significantly suppressed the increased levels of the p-p65/p65 and p-I*κ*B*α*/I*κ*B*α* ratios induced by IL-1*β* activation (Figures 4(b) and 4(c)). Furthermore, immunofluorescence staining showed that IL-1*β*-induced p65 translocation into the nucleus was blocked by pretreatment with 200 *μ*M MA (Figure 4(d)). These results demonstrated that MA suppressed IL-1*β*-induced NF-*κ*B signaling activation in rat chondrocytes *in vitro*.

### 3.4. Protective Characteristics of MA in an OA Rat Model

**Figure 5:**
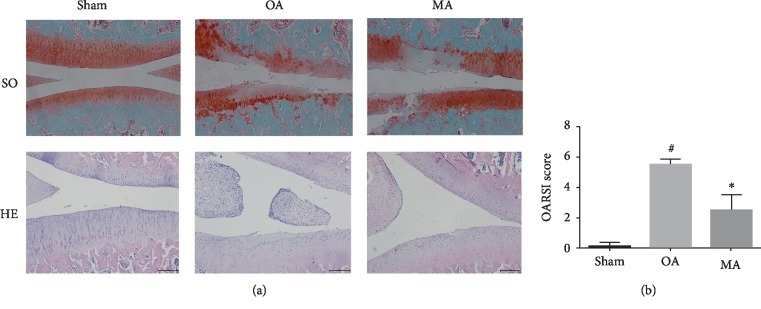
Influences of MA on cartilage degradation in a rat OA model. (a) Articular cartilage slices stained with safranin O/Fast Green and hematoxylin-eosin staining; scale bar = 200 *μ*M. (b) OARSI scoring of the sham group, OA group, and MA group. ^#^*P* < 0.05 vs. sham group, ^∗^*P* < 0.05 vs. OA group. MA: madecassoside; OA: osteoarthritis; SO: safranin O; HE: hematoxylin-eosin.

**Figure 6:**
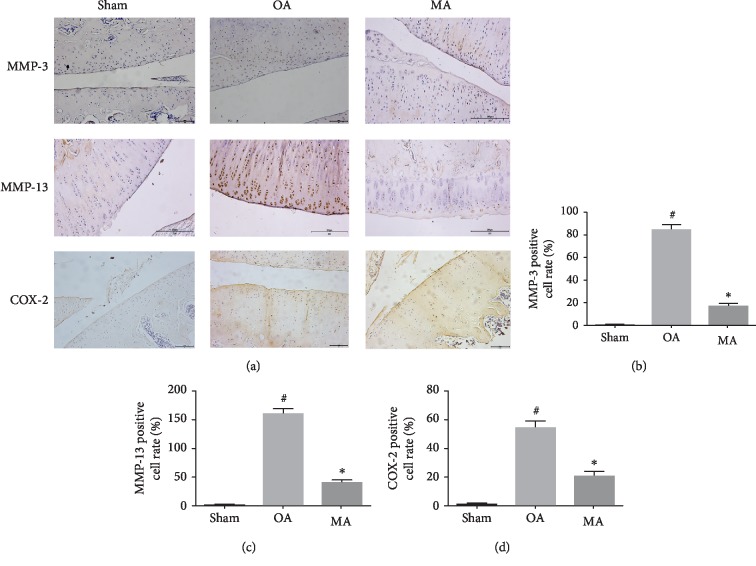
The protective role of MA on cartilage degradation in a rat OA model.Immunohistochemistry for antibody against MMP-3, MMP-13, iNOS, and COX-2 (a), and quantitative analysis (b)–(d). Scale bar = 100 *μ*M/20x. MA: madecassoside; OA: osteoarthritis; MMP: matrix metalloproteinase; COX-2: cyclooxygenase-2.

To determine its protective role *in vivo*, a rat model of OA was used to evaluate the antiosteoarthritic effects of MA. To develop the OA model, a total of 14 rats received surgical resection of the medial meniscus and were then randomly divided into OA- and MA-treated groups. In addition, sham surgery was conducted for 7 rats in the normal group. After one week of surgery, MA solution was injected into the knee joint of the rats in the MA-treated group, once a week for a total of 4 weeks. Histological analysis illustrated that the OA group exhibited OA characteristics compared with the sham group, with obvious damage to the articular cartilage structure. However, cartilage degeneration was clearly ameliorated in the MA-treated group, compared with the OA group (Figure 5(a)). OARSI score analysis was used to confirm the protective characteristics of MA (Figure 5(b)). Safranin O staining was markedly increased in the MA-treated group when compared with the OA group; hematoxylin-eosin staining supported the outcomes of safranin O and showed that MA is able to inhibit the hyperplasia of synovium. Immunohistochemistry staining also showed that MMP-3, MMP-13, and COX-2 expression remarkably decreased in the MA-treated group comparing to the OA group (Figures 6(a)–6(d)). Collectively, these data indicated that MA conferred protective effects *in vivo*.

## 4. Discussion

Currently, treatment for OA includes steroids and NSAIDs. These compounds may relieve pain and swelling but are not able to improve the damage to articular cartilage and often adversely affect the gastrointestinal tract and cardiovascular system. Thus, it is necessary to find safe and effective compounds to inhibit cartilage degeneration. In the current study, the possible influences of MA were determined using a rat articular cartilage degeneration model, where the NF-*κ*B pathway was inhibited. Firstly, we found that MA treatment at various concentrations (0, 25, 50, 100, 200 *μ*M) does not have any toxicity role on chondrocyte viability. Furthermore, MA does not alter the expression of MMPs, COX-2, iNOS, SOX-9, and collagen II at the mRNA and protein levels in the absence of IL-1*β*. In addition, we evaluated the role of MA in the absence of IL-1*β* on the NF-*κ*B signaling pathway. Collectively, all these data reflect that MA does not impair chondrocyte viability.

Various studies have demonstrated the MMP-3 and MMP-13 to cartilage degradation in OA [14] and the low expression levels of chondrocyte-specific proteins such as collagen II and sox9 [15, 16]. MMP-3 degrades the extracellular substrates of the cartilage matrix, while MMP-13 is involved in collagen type II degradation [17, 18]. Thus, in the preset study, the expression levels of MMP-3 and MMP-13 were determined to assess damage to the ECM in OA.

Previous reports have demonstrated that IL-1*β* is involved in the progression of OA; the IL-1*β*-induced activation of chondrocytes resulted in increased expression of MMPs [19]. IL-1*β* was able to increase iNOS and COX-2 expression levels [20]. COX-2 and iNOS play a considerable role in the developmental process of OA [21]. Consequently, the suppression of IL-1*β*-induced inflammation upregulated MMPs, iNOS, and COX-2 and presented a potential therapeutic intervention for OA. The present study indicated that MA reduced the IL-1*β*-induced expression of degrading gene and components of the inflammatory response, including MMP-3, MMP-13, iNOS, and COX-2 (Figure 2). Moreover, the expression levels of type II collagen and sox-9 may also be revived by MA-administration (Figure 3). In addition, MA is able to restore the IL-1*β*-induced reduction of safranin O in chondrocyte (Figure 1). Collectively, these outcomes indicate the antidegenerative role of MA with respect to chondrocytes *in vitro*.

The NF-*κ*B pathway serves a vital role in chondrocyte-associated inflammation, MMP regulation, and cartilage degradation [22], which was the focus of the present study. When stimulated by proinflammatory elements such as IL-1*β*, the level of p65 phosphorylation is increased, and phosph-p65 is rapidly translocated from the cytoplasm to the nucleus. Here, p65 upregulates the expression of proinflammatory genes such as MMP, iNOS, and COX-2 [23, 24].

In the present study, western blot analysis demonstrated that MA was able to regulate p65 phosphorylation and NF-*κ*B inhibitor *α*, indicating that NF-*κ*B signaling serves an inhibitory function in chondrocytes (Figure 4). Previous studies have reported that suppression of the NF-*κ*B pathway in osteoarthritic chondrocytes may be a successful method for relieving inflammation-induced cartilage degeneration.

The present study demonstrated that blocking NF-*κ*B signaling with MA in rat chondrocytes resulted in the reduced expression of IL-1*β*-induced inflammatory factors. Additionally, the articular cartilage was evaluated in the animal experiment. In the cartilage of the OA group, an erosion, loss of cartilage matrix, and synovium hyperplasia were observed (Figure 5). The cartilage was alleviated, and the synovium hyperplasia was inhibited in the rats of the MA-treated group, and the immunohistochemistry results showed that MA was able to reduce the expression of MMP-3, MMP-13, and COX-2 *in vivo* (Figure 6). Furthermore, MA treatment decreased the OARSI scores and inhibited activation of the NF-*κ*B pathway in an OA model of mouse. Further preclinical investigations are required to determine the potential of MA as an anti-inflammatory treatment for OA.

## 5. Conclusion

The present study determined that MA suppressed chondrocyte-associated inflammation by regulating NF-*κ*B signaling *in vitro* and attenuated cartilage degeneration *in vivo*. These findings suggest that MA has a therapeutic potential for the treatment of OA.

## Data Availability

The data used to support the findings of this study are included within the article.
